# Effects of Exogenous Auditory Attention on Temporal and Spectral Resolution

**DOI:** 10.3389/fpsyg.2018.01984

**Published:** 2018-10-23

**Authors:** Basak Günel, Christiane M. Thiel, K. Jannis Hildebrandt

**Affiliations:** ^1^Department of Psychology, University of Oldenburg, Oldenburg, Germany; ^2^Cluster of Excellence Hearing4all, University of Oldenburg, Oldenburg, Germany; ^3^Department of Neuroscience, University of Oldenburg, Oldenburg, Germany

**Keywords:** exogenous auditory attention, acoustic scenes, spectral resolution, temporal resolution, frequency change detection, gap detection

## Abstract

Previous research in the visual domain suggests that exogenous attention in form of peripheral cueing increases spatial but lowers temporal resolution. It is unclear whether this effect transfers to other sensory modalities. Here, we tested the effects of exogenous attention on temporal and spectral resolution in the auditory domain. Eighteen young, normal-hearing adults were tested in both gap and frequency change detection tasks with exogenous cuing. Benefits of valid cuing were only present in the gap detection task while costs of invalid cuing were observed in both tasks. Our results suggest that exogenous attention in the auditory system improves temporal resolution without compromising spectral resolution.

## Introduction

Listening to a single person speaking in a crowded room while several other conversations and background sounds are present is a demanding but very common situation. In acoustic scenes likes this, comprising multiple competing sound sources, the auditory system parses complex auditory input into perceptual components and constructs the incoming sound sources into aggregated signals called acoustic streams (Bregman, 1990). Similar to focusing on specific objects of interest in crowded visual scenes, auditory attention helps us to selectively pick out single acoustic streams to listen to (Best et al., 2008; Shinn-Cunningham, 2008). Due to the importance of auditory attention in our day-to-day life, research into both underlying mechanisms and consequences for sensory processing remain areas of high interest.

Attention to auditory streams can either be voluntary and goal-driven (endogenous, top-down), e.g., enabling a listener to follow a single speaker in a multi-conversation environment or exogenously triggered. Exogenous attention is stimulus-driven and involuntary (bottom-up) and results in capturing and orienting of attention toward sudden, salient stimuli (Chun et al., 2011; Awh et al., 2012). Capture may be evoked by potentially important sounds and force attention to specific auditory streams or sources, e.g., “looming” of an approaching object that has been shown to increase the speed and accuracy of source localization (McCarthy and Olsen, 2017). In multi-stream auditory scenes, auditory stimuli that capture attention have been shown to degrade perceptual processing of a target stream (e.g., Schröger, 1996; Dalton and Lavie, 2004, 2007; Dalton and Hughes, 2014). However, attention may not only modulate higher order processes like speech recognition, but also more basic sensory representations of the target stream, and more cognition-related attentional processes may build on this. Thus, in order to understand modulation of high-level processing, it is important to understand how attention and attentional capture influence perception of more basic features of the target sounds. The effect of capturing attention on the representation of more basic features of sound along the auditory pathway has drawn relatively little consideration so far.

The auditory periphery encodes the most fundamental features of sound, that is, the spectral content and temporal modulation. The ability to represent these basic features at high resolution is critical for the parsing of more complex acoustic signals (Feng and Ratnam, 2000; George et al., 2007). Here, we aim to investigate the effect of exogenous attention on temporal and spectral resolution in auditory perception. A deeper understanding of these effects should not only help in the interpretation of attentional modulation of the perception of more complex signals such as speech but is also a prerequisite for insights into underlying mechanisms, which can then be studied in both human and animal models.

Temporal resolution in the auditory system refers to the ability to resolve details of the modulation of sounds. The most common way to assess temporal resolution in both physiology and perception is the detection of short gaps in an ongoing sound. Gap detection performance has been shown to be predictive for the recognition of more complex signals, such as speech (Phillips, 1999; George et al., 2006, 2007). Mechanistically, temporal resolution on the perceptual level is associated with reliable and highly synchronized neuronal activation along the auditory pathway. Spectral resolution on the other hand describes the ability to resolve different frequency components of an acoustic signal, containing essential information about it. For example, both music and speech processing require resolution of their spectrum to be processed and enjoyed, especially in complex environments (Henry et al., 2005; Shannon, 2005; Davies-Venn et al., 2015). Along the auditory system, spectral information is encoded in spatially organized tonotopic maps. Due to the nature of underlying neural representation, precise encoding of temporal and spectral aspects of sound have opposite requirements on neural processing. Temporal resolution is enhanced by the integration across populations, which may degrade spectral information across the tonotopy. Spectral processing on the other hand benefits by integrating over time, thereby degrading temporal precision.

Attention has been shown to selectively modulate sensory processing of different dimensions of acoustic stimuli, including spectral (Greenberg and Larkin, 1968; Scharf et al., 1987; Botte et al., 1997; Cervantes Constantino et al., 2012; Sohoglu and Chait, 2016), temporal (Demany et al., 2017), and spatial (Best et al., 2008; Collins and Schirillo, 2013) features. However, despite the importance of this aspect, surprisingly little is known on the effects of attention on processing of basic acoustic features of sounds in complex acoustic scenes. The few studies that have addressed attentional effects on the processing of basic acoustic features in multi-stream scenes concentrated on endogenous attentional processes (Botte et al., 1997; Cervantes Constantino et al., 2012; Larson and Lee, 2013; Demany et al., 2017). In these experiments, attention enhanced both spectral (Botte et al., 1997) and temporal (Demany et al., 2017) resolution for targets in the attended stream. Whether exogenous capturing attention provides enhanced sensory resolution for the stream that attention is drawn to has not been studied systematically. Since exogenous and endogenous attention have been shown to be supported by different mechanisms and to have different effects on sensory processing (Chica et al., 2013), we here aim to investigate whether exogenous attention has the same effect as seen previously for endogenous attention in the auditory domain.

Studies from the visual domain indicate that there are both benefits and costs to exogenous attention (Henderson and Macquistan, 1993; Carrasco, 2011; Seifried and Ulrich, 2011; Chica et al., 2013). Similar to spectral information in the auditory system, space is encoded in spatial maps along the visual pathway. Exogenous attention increased spatial resolution in vision (Yeshurun and Carrasco, 1999, 2000), but degraded temporal resolution (Yeshurun and Levy, 2003). The authors postulate that the reason for the observed effect is a trade-off between integration across time and space (see above). Thus, based on the studies in the visual domain, we propose that exogenously driven attention affects perceptual resolution of spectral and temporal aspects in both attended and unattended targets in a multi-stream scene. Given that temporal resolution is more crucial for auditory processing than spectral aspects (George et al., 2007), we expect attention to improve temporal while degrading spectral resolution.

To investigate the effects of exogenous cueing on auditory temporal and spectral resolution, we used an acoustic scene composed of two sequences of alternating tone pips. Such simple sequences reliably evoke a two-stream percept (Bregman and Campbell, 1971). Our subjects had to detect targets appearing at random times in either stream. In order to exogenously capture attention toward or away from the target stream, the intensity of the tone pips directly preceding the target was altered – either in the same stream as the target or in the respective other. In the first task, we used a classical gap detection paradigm, which has been commonly used to measure temporal resolution. In the second task, the listeners had to detect changes in the frequency of the tone pips, a measure commonly used to test spectral resolution.

## Materials and Methods

### Subjects

Eighteen subjects (nine females and nine males between the ages of 18 and 35 years) participated in the study. All participants were right-handed and had pure-tone hearing thresholds of 20 dB HL or less between 125 and 8000 Hz. Ethics approval was obtained from the local ethics committee (Kommission für Forschungsfolgenabschätzung und Ethik, No: 56/2016). The study was conducted in accordance with the Declaration of Helsinki, and all procedures were carried out with the adequate understanding and written informed consent of all participants. All subjects received a monetary compensation for their participation.

### Procedure and Tasks

#### Auditory Calibration Measurement

Before the start of the experiment, we applied an auditory calibration measurement using a sound level meter. For this measurement, the reference dB values for the major frequencies used in the experiment (streams of 750 and 1500 Hz tone pips) for both the left and the right ear were obtained using Brüel & Kjaer Artificial Ear Type 4153 and Hand-Held Analyzer Type 2250/2270. These reference values were then used to adjust stimulus presentation to 60 dB HL on an individual level.

#### Experiment

**FIGURE 1:**
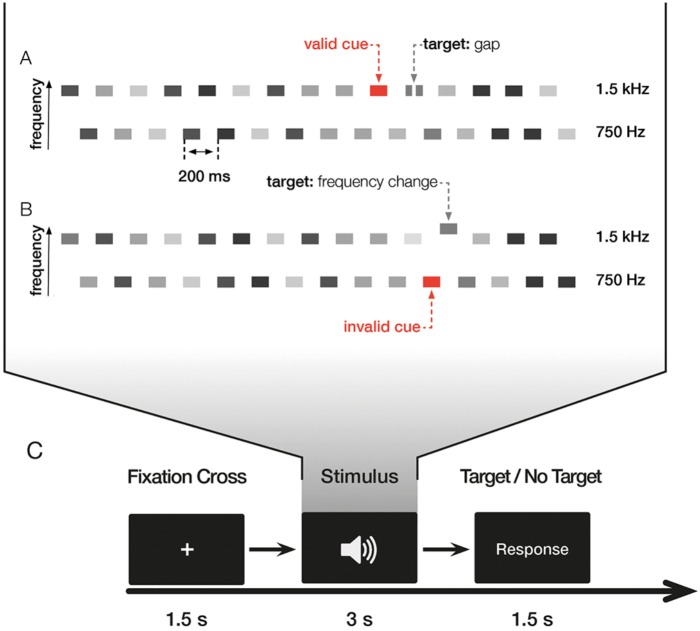
Illustration of the experiment. The experiment consisted of two different tasks with valid trials, where the cue and the target were in the same stream, invalid trials, where the cue and the target were in different streams, and uncued trials with no cue. **(A)** In the gap detection task (GDT), the target was the gap; in the example shown here, the target occurs in a valid trial. **(B)** In the frequency change detection task (FCDT), the target was the change of the frequency; in the example shown here, the target occurs in an invalid trial. **(C)** Illustration of the trial. Both tasks were presented in four blocks consisting of 288 trials in total. Each trial started with a fixation cross followed by the auditory stimulus, i.e., the sequence of interleaved tone pip trains, presented binaurally via headphones. Subjects had to decide whether a target (i.e., gap in GDT and frequency change in FCDT) was present (middle finger of the right hand) or absent (index finger of the right hand) at the end of each trial by button presses. 50% of trials contained a target.

The experiment consisted of two similar tasks investigating the effects of exogenous cueing on temporal and spectral resolution, respectively (see Figure 1). In the first task, subjects had to detect gaps within a tone pip in one of two streams of tone pip trains (gap detection task, GDT) and in the second task, subjects had to detect an upward change of the frequency of a tone pip in one of two streams of tone pip trains (frequency change detection task, FCDT). Both tasks were presented in four blocks of 288 trials in total and followed each other, lasting approximately 60 min. Task order was counter-balanced across subjects to reduce the influence of order effects.

Each trial began with a fixation cross of 1500 ms followed by the tone pips for 3000 ms (Figure 1). Subjects were instructed to decide after the end of each sequence whether it contained the respective target (i.e., gap or frequency change) or not with the middle/index finger of the right hand, respectively. 50% of the trials contained a target, subjects were informed that trials may or may not contain a target. The interstimulus interval (ISI) between two trials was 1500 ms. 50% of the trials contained a target. Auditory stimuli were presented binaurally via AKG K-240 MKII headphones.

#### Stimuli and Conditions

In both tasks, a stimulus consisted of two sequences of interleaved pure tone pip trains. Pure tone pips were presented at 5 Hz repetition rate. Each tone pip had duration of 100 ms including 5 ms cosine ramps at the beginning and end with a frequency of 750 and 1500 Hz, respectively. There were 15–30 tone pips of each frequency per stimulus. The fast repetition rate and large frequency separation ensured a clear two stream percept (Bregman and Campbell, 1971). All stimuli were generated using MATLAB software (Mathworks, Inc., Natick, MA, United States).

Table 1 shows all the conditions. Exogenous cueing was achieved in both tasks by setting the level of the pip train prior to the target to 6 dB above mean background level. 50% (72/144) of total trials contained targets. In 24 (16.7%) of these trials, the cue and the target were in the same streams (valid trials) and in 24 (16.7%) of the trials, the cue and the target were in the different streams (invalid trials). 48 (33.3%) of the trials consisted of the uncued condition. The conditions for both tasks were pseudorandomized across subjects.

**Table 1:** Numbers of trials in each condition used in each task.

	Target	No target
Valid cue	24	48 (cue in either stream)
Invalid cue	24	
No cue	24	24

#### Task 1: Gap Detection Task

Gaps were introduced into a single tone pip in one of the two streams randomly between 2000 and 3000 ms within a trial. The gap duration was either 16 or 32 ms. Eight subjects performed the task with a gap duration of 16 ms whereas 10 subjects performed the task with a gap duration of 32 ms. Duration of the gap was based on pilot experiments and previous literature (Horváth and Winkler, 2010).

#### Task 2: Frequency Change Detection Task

The tone sequences in this task were the same as for the stimuli in the gap detection task. In order to avoid that subjects solved the task based on loudness instead of frequency changes, we introduced level roving to the background stream. Level roving was set to 4 dB and the cue was 6 dB louder than the baseline mean level. The amount of level roving was chosen in pilot experiments as a compromise to mask loudness effects without breaking the clear streaming percept. It is in a range typically used in similar experiments (Moore and Glasberg, 1989; Sussman et al., 2007). Frequency change was 16% for all subjects. The value for frequency change was chosen to be above threshold in pilot experiments and to allow comparison to an animal study that was carried out in parallel.

### Statistical Analysis

Statistical analysis was performed with SPSS23. Based on the application of the signal detection theory (SDT) in a wide range of psychophysical studies on humans and animal models (Green and Swets, 1966; Yeshurun and Levy, 2003; Luo et al., 2007; Alves-Pinto et al., 2012; Engell et al., 2016; Cervantes Constantino and Simon, 2017), we used the sensitivity index *d*′ to calculate the performance measures, which allowed us to take the different strategies of the subjects into account as well as a better comparison of performance in the two tasks independent from a possible change in decision rules. In the context of our study, a higher *d*′ represents a better performance in distinguishing between target (gap or change of frequency) and no target; we were particularly interested in the effect of exogenous cueing on the subjects’ detection sensitivity. The sensitivity index *d*′ was computed using MATLAB software for each subject and each condition (valid, invalid, and uncued) with the formula *d*′ = *z*(hit rate) −*z*(false alarm) (Macmillan and Creelman, 1991), which indicates the z-transformed probabilities of hit and false alarm rates. Based on the previous studies which applied SDT, the *loglinear* correction was used to adjust the hit rates and false alarm rates, whereby 0.5 was added to the number of hits and the number of false alarms, and 1 was added to both the number of signal trials and the number of noise trials (Hautus, 1995; Stanislaw and Todorov, 1999; Lopez-Poveda et al., 2010; Lago et al., 2015; Jerger et al., 2017). This approach was used to take the non-finite values into account, that is, the extreme values of hit rates and false alarm rates.

Performance differences were assessed by repeated measures analysis of variance (ANOVA) with *cueing* (valid, invalid, and uncued) and *task* (FCDT, GDT) as within-subject variables and *gap* (16 and 32 ms) as between subject factor. A Huynh-Feldt correction was used for violations of sphericity (ε > 0.75). Significant effects were tested *post hoc* using paired samples *t*-tests using Bonferroni adjusted alpha levels of 0.0125.

## Results

**FIGURE 2:**
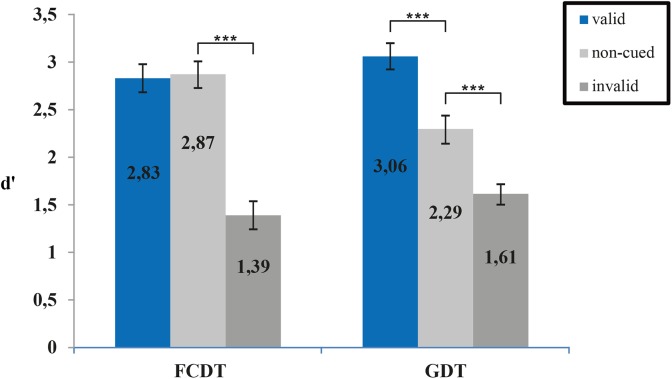
Mean sensitivity indices (*d*′) for invalid, uncued and valid trials in the two tasks testing for spectral (FCDT) and temporal (GDT) resolution. Valid cuing significantly improved gap detection but not detection of a frequency change (^∗∗∗^*p* < 0.001 *post hoc t*-tests).

Overall accuracy was 82% in the FCDT and 79% in the GDT (75% in the 16 ms condition and 82% in the 32 ms condition). In both tasks, cueing affected detection sensitivity *d*′ (ANOVA main effect of *cueing*, *F*_(1.8,28.8)_ = 92.17, *p* < 0.001) with highest detection in the valid cueing condition. There were no differences in detection sensitivity between the two tasks (main effect of *task*, *F*_(1,16)_ = 0.076, *p =* 0.786) but cueing affected detection sensitivity differently in the two tasks (ANOVA *cueing x task interaction F*_(1.56,24.92)_ = 25.56, *p* < 0.001). No significant effects of gap length were found (ANOVA main effect of *gap*, *F*_(1,16)_ = 0.72, *p* = 0.41) nor any further interactions (all *p*-values > 0.5). Figure 2 shows the *d*′ means of the three conditions (valid, uncued, and invalid) for the FCDT and GDT to illustrate the significant cueing x task interaction. *Post hoc* paired samples *t*-tests revealed significant costs of invalid cueing in the frequency detection task (*T*(17) = 11.18, *p* < 0.001) without any benefit of valid cuing (*T*(17) = 0.33, *p =* 0.745). In contrast, in the gap detection task, a clear benefit of valid cueing was found (*T*(17) = −4.87, *p* < 0.001), as well as costs for invalid cueing (*T*(17) = −4.26, *p* < 0.001). Hence auditory exogenous valid cueing improves temporal but not spectral resolution while invalid cueing similarly decreases temporal and spectral resolution.

## Discussion

The present study aimed to investigate the effects of exogenous attention on temporal and spectral resolution in two-stream tasks in normal-hearing adults. To draw exogenous attention toward or away from the stream containing the target, a salient cue was presented just before the target either in the same or the respective other stream. Overall, our results suggest that exogenous attention does affect both temporal and spectral resolution and that effects of exogenous attention depend on whether cue and target are in the same or in different streams. For both tasks, there was a higher performance in trials, for which cue and target were in the same stream compared to trials with invalid cues (Figure 2). Contrary to results from the visual domain (Yeshurun and Levy, 2003), temporal resolution was enhanced by salient cues in the target stream. These results provide evidence that salient cues are able to draw attention toward one out of two competing auditory streams, increasing detectability of targets in the cued stream.

We observed that the valid cue enhanced the subject’s performance in GDT but not FCDT task when compared to the uncued condition. The most likely reason for this difference is a ceiling effect in the FCDT where participants showed high performance in the uncued condition. We used two different gap durations for the GDT where eight subjects performed the task with 16 ms gap length and 10 subjects performed the task with 32 ms gap length. Although there was no significant effect of gap duration on the performance measures for the GDT, it may generally be advantageous to have more precise procedures to determine thresholds on an individual level prior to the full experiments.

Our results for the GDT suggest that valid cues exogenously draw attention toward one stream, increasing target salience in the cued channel and decreasing it in the respective other. Could our results also be explained by simple interactions of cue and target that do not require streaming, namely forward masking or purely temporal interaction (i.e., attentional blink)? Forward masking occurs when a preceding stimulus hinders the ability to respond to the following stimulus within the same auditory filter (Moore, 1998). It typically last up to 200 ms after offset of the masker (Ries et al., 2008; Olsen and Stevens, 2012). This time window would include the onset (100 ms after cue offset) and gap position of the tone pip in the valid trials (cue and target in the same auditory filter). It would thus hamper the detection of the target, not increase its saliency, making it unlikely that forward masking played a role in our experiments. “Attentional blink” refers to the phenomena that a salient stimulus hampers detection of subsequent targets. It has been studied extensively in the visual domain (Dux and Marois, 2009), but has also been found in audition (Mondor, 1998). The attentional blink is strongest for targets immediately following salient cues and quickly decays afterward. In our experiments, invalid cues and targets were separated by 100 ms, while valid cues and targets were separated by 200 ms. Thus, the difference between valid and invalid trials could partially be explained by the attentional blink, irrespective of streaming. However, in a paradigm very similar to ours, but with a single stream of pure-tone pulses, the auditory blink has been shown to extend to at least 270 ms after cue onset (Horváth and Burgyán, 2011). In our experiments, we observed an increase in target salience in the valid trials compared to the no-cue condition, which cannot be explained by the attentional blink, providing evidence for attentional capture beyond a pure attentional blink.

Our study was inspired by evidence from the visual domain, where exogenous attention has been shown to enhance spatial but lower temporal resolution (Yeshurun and Levy, 2003). The authors reason that the underlying mechanism is a trade-off between temporal summation enhancing spatial resolution and the resolution of finer temporal details. In contrast, we report that exogenous attention in the auditory domain *enhances* temporal resolution. Similar to spatial location in the visual system, frequency in the auditory pathway is represented in tonotopic maps. In principle, the same trade-off between temporal and spectral resolution could have been expected in our experiments. This indicates that exogenous attention in the visual and auditory system may be driven by different mechanisms. The simplicity of the task we present provides the opportunity to use animal models to answer questions about the neural underpinning of sensory encoding and attentional modulation. Such studies could give valuable insights into mechanisms behind both regular function and impairment of attentional control in complex scenes.

Since higher order processing relies on the resolution of basic auditory features (Feng and Ratnam, 2000; George et al., 2007), reports on attentional effects on more complex streams, including speech, could partly be explained by changes to the underlying sensory representation at earlier stages of the auditory pathway. Hearing impaired subjects often have severe difficulties in crowded auditory scenes, specifically to focus and keep attention to single speech streams in complex backgrounds. Previous findings already demonstrated a decline in performance measures in spectro-temporal processing and understanding speech for hearing-impaired subjects compared to controls (Larsby and Arlinger, 1999; Grimault et al., 2001; Rose and Moore, 2005). Here, we present evidence for further degradation of both spectral and temporal resolution in the presence of salient, attention-capturing stimuli outside the target stream. Such attention-capturing stimuli could be particularly problematic for hearing impaired subjects, since it may add to their already lower resolution and thereby impair speech recognition particularly in complex environments.

## Conclusion

Although prior research in the visual domain was able to show the effects of peripheral pre-cueing on temporal resolution, surprisingly little was known about the effects of exogenous attention on temporal and spectral resolution in the auditory modality. To our knowledge, this study is the first to provide evidence of the effects of exogenous attention on both temporal and spectral resolution in normal-hearing adults. Our data demonstrate that cues capturing attention in complex scenes enhance both temporal and frequency resolution. Differential modulation of temporal resolution in the visual and auditory domain suggests that different mechanisms by which attention modulates sensory processing reflect the specific demands of each modality. Given the importance of temporal and spectral resolution in understanding speech as well as auditory processing, these effects likely play an important role in navigating complex and multi-speaker environments. Future research elucidating underlying mechanisms in both human and animal models may help to better understand both mechanisms and deficits of auditory scene analysis.

## Author Contributions

KH and CT conceptualized and supervised the study. BG collected and analyzed the data, prepared the figures and wrote the manuscript. All the authors were involved in the design of the study, interpreted the data, provided feedback, and revised the manuscript.

## Conflict of Interest Statement

The authors declare that the research was conducted in the absence of any commercial or financial relationships that could be construed as a potential conflict of interest.
